# Reliability and construct validity of the Instrument to Measure the Impact of Valve Heart Disease on the Patient's Daily Life

**DOI:** 10.1590/1518-8345.0624.2730

**Published:** 2016-12-19

**Authors:** Daniela Brianne Martins dos Anjos, Roberta Cunha Matheus Rodrigues, Kátia Melissa Padilha, Rafaela Batista dos Santos Pedrosa, Maria Cecília Bueno Jayme Gallani

**Affiliations:** 1 Master’s student, Faculdade de Ciências Médicas, Universidade Estadual de Campinas, Campinas, SP, Brazil.; 2 PhD, Full Professor, Faculdade de Enfermagem, Universidade Estadual de Campinas, Campinas, SP, Brazil.; 3 PhD, Associate Professor, Faculdade de Enfermagem, Universidade Estadual de Campinas, Campinas, SP, Brazil.; 4 Doctoral student, Faculdade de Enfermagem, Universidade Estadual de Campinas, Campinas, SP, Brazil.; 5 PhD, Full Titular, Faculté des sciences infirmières, Université Laval, Québec, QC, Canada.

**Keywords:** Sickness Impact Profile, Heart Valve Diseases, Nursing

## Abstract

**Objective:**

evaluate the practicality, acceptability and the floor and ceiling effects, estimate the reliability and verify the convergent construct's validity with the instrument called the Heart Valve Disease Impact on daily life (IDCV) of the valve disease in patients with mitral and or aortic heart valve disease.

**Method:**

data was obtained from 86 heart valve disease patients through 3 phases: a face to face interview for a socio-demographic and clinic characterization and then other two done through phone calls of the interviewed patients for application of the instrument (test and repeat test).

**Results:**

as for the practicality and acceptability, the instrument was applied with an average time of 9,9 minutes and with 110% of responses, respectively. Ceiling and floor effects observed for all domains, especially floor effect. Reliability was tested using the test - repeating pattern to give evidence of temporal stability of the measurement. Significant negative correlations with moderate to strong magnitude were found between the score of the generic question about the impact of the disease and the scores of IDCV, which points to the validity of the instrument convergent construct.

**Conclusion:**

the instrument to measure the impact of valve heart disease on the patient's daily life showed evidence of reliability and validity when applied to patients with heart valve disease.

## Introduction

Chronic disease may begin as an acute condition and extending through episodes of exacerbation and remission of symptoms. While it is possible to control the accumulation of events, the constraints imposed by the treatment can lead to a drastic change in the lifestyle of the subjects ^1^ ^-^ ^2^. Among the chronic diseases that evolve with these features, the cardiovascular disease (CVD), is an important cause of morbidity and mortality in the context of global health^3^. In Brazil, in 2013, cardiovascular diseases were the leading cause of death among all other conditions, corresponding to 28.0 % of the proportion of deaths with defined causes, wit acute myocardial infarction (AMI) being the cause of death of 85,939 people, and of these, 40,366 from the Southeast region. It is worth to highlight that in June 2015 alone, ischemic heart disease accounted for 2.3% of total admissions by the Brazilian Unified Health System^4^.

Valve disease in Brazil, represents a significant portion of hospitalizations for CVD. Rheumatic fever is the main cause of valve disease in the national context, responsible for up to 70% of cases, unlike what happens in developed countries. However, national data on rheumatic fever, obtained through DATASUS, refer to the number of hospitalizations and valvular interventions, which may underestimate the actual number of cases, since it does not include heart valve disease patients diagnosed in the outpatients' clinic and that did not required hospitalization^4^ ^-^ ^5^. The patient with valve disease may have different signs and symptoms (pain, fatigue, arrhythmia, palpitations, syncope, weariness and angina) whose frequency and intensity are associated with the valve apparatus affected, being the mitral or aortic valve , the type of injury (stenosis or insufficiency) and the stage of evolution of the valvular heart disease^5^. Due to the slow and progressive nature of many of these lesions, patients may not recognize the symptoms, since the limitation of their daily activities also occur gradually^6^.

The symptoms experienced by these patients result in changes in physical function, self-esteem, body image, social relations and a number of daily activities^7^ ^-^ ^8^. Therefore, nurses should include in the care of these patients, not only biological aspects, but also those related to psychosocial variables, helping to overcome its limitations and the acquisition of coping mechanisms^9^.

In order to support nursing interventions that minimize the impact of valvular disease in the life of the subject, a specific instrument to measure the perception of valvular disease on the impact of the disease in his life, called "Heart Valve Disease Impact on daily life - IDCV", built and validated by the Brazilian cultural context^10^ ^-^ ^11^.

This instrument showed reliable and valid psychometric properties when applied to valvular disease patients^11^. Although the instrument has been developed to be used with these patients, the refinement of its items resulted in the selection of issues that were pertinent to the impact assessment of valvular heart disease and also to assess the impact on other heart diseases with similar symptoms. In previous studies performed^12^ ^-^ ^15^ the reliability and validity of the instrument were shown, when applied in patients with coronary artery disease, heart failure and hypertension (AHT). A broader study was designed to assess the responsiveness of the instrument when applied to valvular disease patients and cardiac patients undergoing surgery^16^.

However, considering the psychometric evaluation of the performance of IDCV with patients with valvular disease, there was no estimation of the reliability of the instrument with regard to the stability of measurement, just as the occurrence of ceiling and floor effects. Moreover, it was considered important to investigate the construct convergent validity through correlational evidence with a general question regarding the impact of the disease constructed by the authors of IDCV. 

Thus, this study aimed to: assess the feasibility, acceptability and ceiling and floor effects; estimate the reliability with respect to the stability of the measure and verify the convergent construct validity of the Heart Valve Disease Impact on daily life mitral valve disease and / or aortic outpatients at a university hospital. 

The results of this study contribute to the psychometric refinement of an instrument built in the Brazilian culture in order to measure the impact of valvular disease in subjects' lives. The availability of a reliable, valid and robust tool can guide health professionals in the development of more effective behaviors to minimize the impact of disease on daily life of these patients.

## Methods

### Type of study

This is a methodological instrument validation study to measure the impact of the disease - the IDCV^17^.

### Study Location

The study was conducted in the cardiology outpatient clinic - subspecialty valvular disease - of a large university hospital in the interior of São Paulo State.

### Subjects and Sample Size

This study included patients with mitral and / or aortic valve disease, of both sexes, aged over 18 years, subject to clinical and / or surgical treatment, in the aforementioned outpatient clinic. Patients who presented inability for effective verbal communication due to neurological or psychiatric changes were excluded.

The size of the sample followed the recommendations of validation studies, i.e., 100 subjects^18^. However, due to losses in the data collection stage, especially in the repeat test step, the final sample consisted of 86 patients.

### Data Collection

Data was collected through personal interviews carried out individually by the researcher to obtain socio-demographic and clinical data, and by telephone to obtain data on the impact of the disease through the application of IDCV. The following data collection steps were established:

- First step: the initial approach to clarify the objectives of the study and obtain the patient's consent to participate by signing the Terms of Free and Informed Consent (TFIC). The patients were instructed that their participation in the study would involve a questionnaire through two telephone contacts. Obtaining consent, it used the data recording method available to obtain information from medical records on the socio-demographic and clinical characterization of subjects studied. Further, through structured interview techniques were obtained socio-demographic and clinic data, not available in the clinical hospital records;

- Second step: We performed the first telephone contact for the application of IDCV (test);

- Third step: 7 to 22 days after the first application of IDCV (test) a new telephone contact was conducted for the second application of this instrument (repeat test). The interval between the test and repeat testing was determined in accordance with the recommendation that the period between applications should not be too short, - to prevent the memory of the responses provided in the first interview - not too long, since the occurrence of other events in the daily lives of patients could explain the variations identified in the scores^(19)^. It is noteworthy that the implementation of IDCV through telephone contact was chosen, because of the difficulties of the participants attending the second time of application of IDCV (repeat test). The literature recommendations for the use of the telephone contact in data collection were followed. In this sense, it was evidenced an invariance in performance scales used in different application groups - in person or by phone, suggesting that telephone contact is a reliable strategy to obtain data when compared to face-to-face interview, in addition to being effective, inexpensive and accessible^(20)^. Thus, in this study the IDCV applications in the test and repeat test were conducted by telephone contact in order to maintain the same method of data collection and avoid bias collection, thus ensuring the accuracy in obtaining these data.

### Data Collection Instruments

*- Socio-demographic and clinical instrument:* It was used an instrument built and submitted to content validity^(21 )^;

a) Socio-Demographic Data: to obtain data such as: name initials, age, registration number in the chart, gender, education, marital status, employment, with whom they live, family and individual monthly income;
b) Clinic Characterization: Individuals data were collected for signs and symptoms that occurred during the month immediately prior to the collection and by consulting the records, information was obtained about the date of diagnosis of valvular heart disease, type of valve disease, treatment data (medical or surgical) and medicines in use.

*- Heart Valve Disease Impact on daily life (IDCV)* ^10^ ^-^ ^11^: developed and validated in order to evaluate the impact of valve disease in the patient's life. This is an instrument consisting of two scales (A and B). Part A has items with statements regarding the impact, and Part B items assessing each of the consequences listed in Part A, for a total of 14 items. The items are grouped into four factors or domains: *Physical impact of the disease - symptoms*- (Items 11, 12 e 13); Impact of the disease on daily activities, (5, 7, 9, 10 and 14); *Social and emotional impact of the disease*, (Items 2, 3, 4 e 6) and A*daptation to illness* (1 and 8). In the first scale (Part A) the Likert response scale of five points is used, from (1) strongly disagree to (5) strongly agree. In Part B, which measures the assessment that the subject has on each result of valvular disease mentioned in the first scale (Whether it occurs or not in his/her life), the answers range from (1) very bad to ^5^ very good. To calculate the score, each item corresponds to the product of the scores obtained in Parts A and B of IDCV, generating a minimum score of 1 and a maximum of 25 for each statement evaluated. The closer the score is to 1, the lower the impact felt by the subject, and the closer it is to 25, the greater the impact. In Part A, which measures the intensity of agreement / disagreement with the statements, items 1, 5 and 8 for addressing the favorable impact perceptions, have their score inverted so that all statements can be assessed in the same direction, therefore, the higher the score the greater the impact. In Part B, the scores of all items are reversed and, the lower the score, the better the evaluation that the patient makes of the affirmative. The final score of the measured impact is obtained by summing all the products, with a possible variation score of 14-350. The lower the score, the less the patient perceives the negative consequences of the disease in his/her life evaluating them as bad. On the other hand, the higher the score, the more the patient recognizes the occurrence of the negative consequences of the disease in his life, and these consequences, in fact, are interpreted as negative. The IDCV yet has a general question of impact assessment (which is not included in the calculation of the total score) - "When considering all the consequences of heart disease in your life, how do you assess the impact of the disease?" with scores ranging from 1. Very bad 2. Bad 3. Do not know the answer 4. Good and 5. Very good; the lower the score the greater the impact of the disease. In the present study, this question was considered a general measure of the impact of the disease and used to test the convergent construct validity of IDCV. Although IDCV was originally developed for evaluation of beliefs from valvular disease patients, it appears that the set of statements that compose it assesses the impact of chronicity imposed by different cardiovascular diseases^11^. The instrument obtained satisfactory psychometric performance when applied in CAD ​​patients^12^ ^-^ ^13^, those with heart failure^14^, just as in hypertensive patients^15^. 

### Data Analysis

The psychometric properties of IDCV were assessed according to the criteria recommended by international literature^22^. Data were entered into a spreadsheet (Excel Software, 2010) and transferred to SPSS - Statistical Package for Social Sciences, version 17.0 for Windows, for the following analysis:

*- Descriptive:* frequency tables, position measurements (mean, median, minimum and maximum) and dispersion (standard deviation) for data of socio-demographic and clinical characterization instruments. The *practicality* of the IDCV was evaluated by the time for instrument application, and the time of the interview as clocked by researcher. The feasibility / acceptability was assessed by the percentage of unanswered items and the proportion of patients who did not respond to all items^22^. To analyze the ceiling effect the percentage of patients who rated ceiling, i.e. 10% showing the highest scores IDCV (indicating greatest negative impact of disease)^23^ was calculated, both for the total IDCV (≥316.4) as for its domains (physical impact of the disease - symptoms ≥67.8, impact of disease on daily activities ≥113.0, social and emotional impact of illness and adaptation ≥90.4 disease ≥45.2). It was also estimated the percentage of patients who rated floor^23^, that is, showed 10% of the smallest possible scores IDCV (Therefore, the lowest scores, which means less impact - ≤10.2 symptoms, disease impact ≤17.0 in daily activities, social and emotional impact of the disease ≤13.6 and adaptation to the disease ≤6.8). Ceiling and floor effects were considered moderate up to 25% and substantial when higher than 25%^23^.

- *Reliability* with regards to the agreement between repeated measurements (Test- repeat test) through the use of the intra-class correlation coefficient (ICC). Coefficient ICC > 0.70^24^ was considered as evidence of measurement stability. 

- *Convergent construct validity* through the use of the Spearman correlation coefficient to verify the relationship between domain scores and total IDCV and the score obtained by applying the general question on the impact of the disease provided by IDCV "When considering all the consequences of heart disease in your life, how do you assess the impact of the disease". The following criteria were used to classify the magnitude of correlations: Correlations <0.30 were considered of low magnitude, between 0.30 and 0.50 for moderate and> 0.50 strong magnitude^25^.

The significance level for the statistical tests was 5%.

### Ethical Aspects

The study was approved by the Ethics Committee of the local university through an addendum to broader project developed in the research group (Resolution nº 843/2010). Patients involved signed the TFIC. 

## Results

### Socio-demographic and Clinical Characterization

Of the 86 participants, 58.1% were women, with a mean age of 52.7 (SD = 12.9) years, average schooling years of 6.4 (SD = 3.2) years; 55.8% were married, living with a spouse and children (41.9%), inactive (47.7%), with average individual income of 1.9 (SD = 1.2) minimum wages (MW) and household income average of 2.9 (SD = 1.9) MW month.

Considering the clinical diagnosis, 37.2% of patients have a single lesion in a single valve device and 31.4% of subjects showed involvement of more than one valve apparatus. Fatigue (53.5%) and dyspnea (50%) were the most frequently reported symptoms. The median time from start of treatment was 14.1 (SD = 12.6) years. Most patients (71.3%) were in clinical treatment and had undergone surgical treatment. These patients consume an average of 4.4 (SD = 2.1) types of medication a day.

### Analysis of the feasibility, acceptability and ceiling and floor effects of IDCV

All patients responded in full to all items of IDCV, pointing to high acceptability test in the sample. The practicability of IDCV assessed with patients with valvular heart disease, was tested by time spent in the application of the instrument, being observed an average time of 9.9 (SD = 3.3) minutes, ranging from 4.7 to 27.1 minutes. The application time was measured by the researcher with the aid of a digital timer that allowed measurement of tenth of minutes.

Regarding the analysis of the ceiling and floor effects (Table 1), it is pointed out that 32.6% of patients scored in the floor area of Adaptation to the Disease and 17.4% in the area called Physical Impact of the Disease - Symptoms. Regarding the ceiling effect, 11.6% of patients scored in 10% of the highest scores of the scale in the field of Physical Disease Impact - symptoms, i.e., scores indicating greater impact of the disease in the subject's life.

**Table 1:** Descriptive analysis and ceiling and floor effects of the instrument Heart Valve Disease Impact on daily life (IDCV) in heart valve disease patients (n=86) Campinas, SP, Brasil, 2012.

Field	Items	Average (dp)	Median	Amplitude	Definition Floor effect*	Definition Ceiling Effect†	Floor Effect (%)	Ceiling Effect (%)
IDCV^‡^ total	14	154.5 (70.4)	152.5	301.0	Scores	Scores	8.1	1.2
					≥316.4	≤47.6		
Physical Impact - symptoms	3	35.8 (21.6)	34.0	72.0	Scores	Scores	17.4	11.6
					≥67.8	≤10.2		
Disease's Impact - Daily activities	5	62.3 (31.8)	65.5	120.0	Scores	Scores	9.3	3.5
					≥113.0	≤17.0		
Social and Emotional Impact of the disease	4	43.1 (25.2)	41.0	89.0	Scores	Scores	12.8	1.2
					≥90.4	≤13.6		
Adaptation to the Disease	2	13.4 (8.7)	11.0	34.0	Scores	Scores	32.6	--
					≥45.2	≤6.8		

*Floor effect equivalent to 10% of the lowest scale scores; †Ceiling effect equivalent to 10% of the highest scores of the scale; ‡IDCV - Heart Valve Disease Impact on daily life.

### Reliability Analysis

To evaluate the reliability of the instrument we considered the criteria of temporal stability with use of test and repeat test. The total of 70 patients responded to the repeat test. It has been found adequate degree of agreement in the estimates of intra-class correlation coefficients (ICC) for the total IDCV and for most areas, and found less ICC in the field of Disease Impact on Daily Activities (ICC = 0.76), as shown in table 2.

**Table 2:** Intra-class correlation coefficients (ICC) and confidence intervals (95% CI) of the instrument Heart Valve Disease Impact on daily life (IDCV) when applied to heart valve disease patients (n = 70). Campinas, SP, Brazil, in 2012.

IDCV*	ICC	CI‡ 95%
Physical Impact of the disease - symptoms.	0.77	0.67-0.86
Impact of the disease in daily activities.	0.85	0.79-0.92
Social and Emotional impact of the disease.	0.85	0.79-0.92
Adaptation to the disease.	0.81	0.73-0.90
IDCV Total.	0.91	0.87-0.95

*Heart Valve Disease Impact on daily life - IDCV; †ICC=Intra-class Correlation Coefficients; ‡CI=Confidence Intervals.

### Analysis of convergent construct validity

The results of the convergent construct validity assessed by the correlation between the total scores and the areas of IDCV and the scores of the general question on the assessment of the impact on subject's life are presented in Table 3.

**Table 3:** Spearman correlation coefficients between the score of the generic question about the impact of disease and IDCV global and domains scores*. Campinas, SP, Brazil, in 2012.

Variables	1	2	3	4	5	6
IDCV^*^ Physical - Symptoms.	1.0					
IDCV Daily Activities.	0.52^†^	1.0				
IDCV Social and Emotional.	0.48^‡^	0.68^‡^	1.0			
IDCV Adaptation to the disease.	0.22^§^	0.36^§^	0.30^§^	1.0		
IDCV Total.	0.73^‡^	0.90^‡^	0.86^‡^	0.46^‡^	1.0	
Generic Measure - Impact.	-0.37^§^	-0.47^‡^	-0.52^‡^	-0.05^†^	-0.53^‡^	1.0

*Heart Valve Disease Impact on daily life - IDCV; †Not significant; ‡ p< 0,0001; §p≤0,0005;

Considering the assessment of the general question of the impact, the lower the score the higher the impact perceived by the subject and that the interpretation of the total score of IDCV the lower the score, the lower the impact perceived by the subject, statistically significant negative correlations were expected between variables analyzed. Negative significant correlations were found, moderate to strong magnitude between the measurement provided by the generic question about the impact of the disease and the total IDCV and most of its domains, except for the Adaptation to Disease that showed no correlation with the generic measure of impact. There was a significant correlation of strong magnitude between the scores of the general question of impact and the total IDCV (*r* = -0.5273), just as among the general measure and the field Social and Emotional Impact (*r* = -0, 5174) (Table 3).

## Discussion

This study aimed to evaluate the feasibility, acceptability and ceiling and floor effects; estimating the reliability as regards the stability of the measure and verify validity of the convergent construct IDCV, when applied to patients with valvular heart disease as outpatients. 

The evaluation of practicability of the IDCV showed that it is a rapid implementation tool with an application average time of 9.9 minutes (SD = 3.3). This finding is consistent with those found in previous study ^12^, in which the average application time was 09 minutes, according to another study^15^, the application of IDCV lasted 08 minutes. Regarding acceptability, all patients responded to all items, not being detected a score 3, which corresponds to the neutral response. Thus, the findings show that the IDCV proved easy to apply in the study group. 

In the present study we evaluated the ceiling effects, which may indicate involvement in the instrument's ability to detect changes in health status with regard to the increase in perceived impact and thus, in situations of clinical worsening. Floor effect was detected suggesting impairment in the instrument's ability to detect changes in situations where there is an improvement in health condition due to the reduced impact of the disease^23^.

The analysis of data on ceiling and floor effects revealed a moderate ceiling effect in the domain *Physical Impact of Disease - symptoms* and substantial floor effect on the domain Adaptation to disease and moderate in other areas of scale, especially in the domains *Physical Impact of Disease* - Symptoms and *Impact social and Emotional disease*. 

In other studies of IDCV, it was also observed floor effect in the areas of *Physical Impact of the Disease - symptoms* and in the field of *Social and Emotional Impact* ^12^ ^,^ ^15^. In a study by Santos et al.^12^, 49.0% of patients scored in the floor area of *Adaptation to the disease.* However, contrary to the findings of the present study, in a previous study, 31.4% of patients scored ceiling in the same domain^15^. These findings can be explained in part by the constitution of their items: Item 1 "After I got heart troubles, did I start to pay more attention to my health?" And Item 8 "Is my sex life the same as before the heart problem?" which can cause double interpretation. Respondents can interpret item 1 as a good or bad result and in item 8 understanding its difficulty may be related to the assessment of the consequences of the disease in sexual life, since the individual may not have any information about the quality of life sexual before the disease. 

Another assessed property was the reliability of the instrument by the criteria of temporal stability by using the test- repeat test. We sought to assess whether in a given time interval, the participants' answers to IDCV showed little variation in the absence of external factors that could affect the perception of the subject on the concept studied^15^. In the present study the temporal stability was investigated in the interval of 7 to 22 days, by telephone contact, a strategy previously used^20^. 

An appropriate level of agreement has been found between test / repeat test for total IDCV and for most areas, with the lowest value of ICC found in the field of disease impact on daily activities (ICC = 0.76). These results coincide with those obtained in previous studies^14^ ^-^ ^15^. In the study among patients with heart failure we observed an ICC> 0.96 for most IDCV fields except for the field *Adaptation to the Disease* ^14^. In the study involving patients with AHT it has been found an ICC> 0.99 for the total IDCV, as well as their domains^15^. These results point to the reliability of IDCV when applied to different samples of patients with cardiovascular diseases.

Considering that the results of this study with regard to testing and repeat testing are mostly similar to other IDCV validation studies, it may be considered that the investigation of the measure stability by phone does not interfere with the quality of the findings. However, it is recommended to carry out new studies, with expansion of the sample in order to contribute to building evidence for the application of the test and repeat test by phone. 

As per previous hypothesis, significant negative correlations were found between the extent provided by general question of burden of disease and the total IDCV and most of its domains, suggesting convergent validity. 

Construct validity estimates the extent to which the scores of a measuring instrument are consistent with hypotheses derived from the concept in measurement. It aims to validate an underlying body of theory as a measurement, and test the hypothetical relations presupposed in this theoretical body^17^ ^,^ ^24^ ^).^ It seems there is no consensus on the number of hypotheses to be verified to ensure adequate validity ^26^ ^).^ The convergent construct validity in turn, relates to the correlation between similar constructs measures^24^. According to Polit^27^ in the absence of a gold standard, assumptions about the correlation between instrument scores and the scores of a measure with which is expected conceptual convergence, are tested. Therefore, the finding of a correlation between the total score of IDCV and the general question of the impact of the disease points to the fitness of its items to the concept that tries to measure, one of the important properties in measuring self-reported instruments.

The present study has limitations related to the small sample size and the fact of not having been employed other generic tools for assessing the impact of the disease on the patient's life. Moreover, the generalization of the findings of this study is limited, since the survey was conducted in a sample of patients with valvular heart disease that were outpatients. However, this study adds important contributions to literature, since it collaborates in psychometric refinement of a tool with evidence of reliability and validity for the study of the impact of the disease, which may be useful in evaluating outcomes of nursing interventions, and at the same way it is contributing to the construction of evidence about the quality of test results and applying the repetition test by phone. 

It is suggested to carry out new studies aimed to investigate the responsiveness of IDCV in patients with different cardiovascular diseases.

## Conclusion

This study concludes that the instrument to measure the Heart Valve Disease Impact on daily life - IDCV is an instrument with good acceptability and easy to apply, but it found the need to review the items that make up the domain of adaptation to the disease. The analysis of the ceiling and floor effects points specifically to the occurrence of the floor effect in different areas of the instrument which may indicate a lower potential of the IDCV to detect changes in clinical improvement of conditions. Reliability was demonstrated with respect to temporal stability and convergent construct validity with generic question of disease impact. The results of this study contribute to the refinement of the psychometric properties of IDCV in patients with different cardiovascular diseases. 
